# Application of New Energy Thermochromic Composite Thermosensitive Materials of Smart Windows in Recent Years

**DOI:** 10.3390/molecules27051638

**Published:** 2022-03-02

**Authors:** Yu-Qin Feng, Mei-Ling Lv, Ming Yang, Wen-Xia Ma, Gang Zhang, Yun-Zi Yu, Ya-Qi Wu, Hai-Bo Li, De-Zheng Liu, Yong-Sheng Yang

**Affiliations:** 1 Hubei Key Laboratory of Biomass Fibers and Eco-Dyeing & Finishing, School of Chemistry and Engineering, Wuhan Textile University, 1 Textile Road, Wuhan 430073, China; fyq2740223768@163.com (Y.-Q.F.); yangming491@163.com (M.Y.); mawenxia20201224@163.com (W.-X.M.); prayerzg2019@163.com (G.Z.); yyz17746642685@163.com (Y.-Z.Y.); wuyaqi07@163.com (Y.-Q.W.); 15971439796@163.com (H.-B.L.); 2 Department of Mechanical Electricity, Wuhan Instrument and Electronic Technical School, Wuhan 430074, China; yeternal2016@163.com; 3 Hubei Key Laboratory of Power System Design and Test for Electrical Vehicle, Hubei University of Arts and Science, Xiangyang 441053, China

**Keywords:** smart windows, thermochromic hydrogels, thermochromic organic materials, energy-saving

## Abstract

Thermochromic smart windows technology can intelligently regulate indoor solar radiation by changing indoor light transmittance in response to thermal stimulation, thus reducing energy consumption of the building. In recent years, with the development of new energy-saving materials and the combination with practical technology, energy-saving smart windows technology has received more and more attention from scientific research. Based on the summary of thermochromic smart windows by Yi Long research groups, this review described the applications of thermal responsive organic materials in smart windows, including poly(N-isopropylacrylamide) (PNIPAm) hydrogels, hydroxypropyl cellulose (HPC) hydrogels, ionic liquids and liquid crystals. Besides, the mechanism of various organic materials and the properties of functional materials were also introduced. Finally, opportunities and challenges relating to thermochromic smart windows and prospects for future development are discussed.

## 1. Introduction

The energy crisis is a major problem threatening human society. In recent years, heating, ventilation and refrigeration account for 43% of the global primary energy consumption [1], and the problem of building energy consumption [2] has attracted widespread attention [3]. In the global total energy consumption, building energy consumption accounts for more than one-third, and nearly half of it is lost by building windows.

At present, to solve the problem of building energy consumption, usually adopt the method of dynamic adjustment [3], changing building roof and metope adornment to achieve the goal of energy saving. For example, the use of highly reflective cooling coatings [4], wet roofing [5] and porous materials [6] not only have certain effects on maintaining indoor temperature stability but also have non-negligible defects. When the weather is cold, the coating will not work, and it is difficult to reduce the building heating energy consumption. The coating is not suitable for year-round use [7]. In addition, the wet roof is not suitable used in the winter [5]. In the complex natural environment, a system that can spontaneously respond to temperature changes to meet the needs of practical applications, in order to better solve the problem of building energy consumption [8].

Compared with the development of an intelligent regulation system on the top of the wall, window renovation is the most effective and simple way to realize the intelligent heat transfer controlling and effectively reducing the total energy consumption of the buildings [9]. Therefore, it is necessary to improve the glass window [10,11]. Generally ideal smart windows usually meet the following conditions: (1) In the use of the process does not increase energy consumption; (2) wide range of regulation and control; (3) strong self-regulation ability of the system.

To solve the above problems, thermochromic smart windows [12] have been researched. Under heating or cooling conditions, the optical transmittance, reflectivity and color of thermochromic materials will change significantly. They are the best materials for making smart windows. Compared with electrochromic smart windows and gas smart windows [13], thermochromic smart windows have the advantages of no external control device, no need to consume additional energy and simple structure [14].

**Figure 1:**
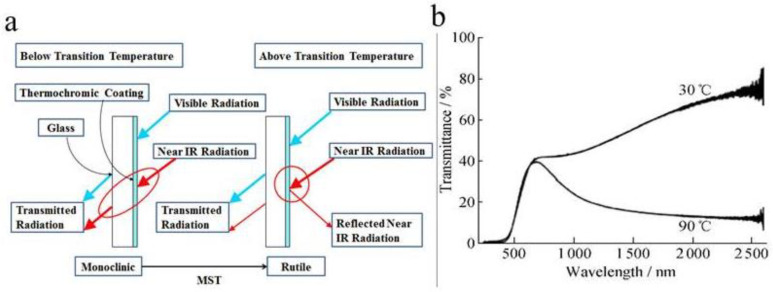
(**a**) Schematic diagram of VO_2_ thermochromic smart window dimming [17]. (**b**) Optical transmittance curves at VO_2_ in high temperature (90 °C) and low temperature (30 °C) states [18].

Thermochromic material-produced smart windows commonly exhibit an inorganic phase change material and an organic polymer material. Inorganic phase change material mainly focuses on vanadium dioxide (VO_2_), which undergoes a metal–insulator transition at the critical temperature (*T*_c_) of 68 °C, accompanied with huge transmittance contrast in the IR range and negligible change in the visible range. Vanadium dioxide (VO_2_) used as the thermochromic material has attracted more attention in recent years [15]. When the external temperature (*T*) is lower than the phase transition temperature (*T*_c_), vanadium dioxide will undergo a reversible phase transition. VO_2_ is a monoclinal phase with low reflectance in the near-infrared band [16]. When the external temperature (*T*) is greater than the phase transition temperature (*T*_c_), the phase of VO_2_ could change from a monocline state to a rutile state (Figure 1a). In this state, VO_2_ has a large number of free electrons, the transmittance of the near-infrared band drops and the reflectivity increases [16]. The reversible transformation of vanadium dioxide from monoclinic crystal phase to rutile crystal phase increases its absorbance in the near-infrared spectrum. The optical throughput curve is shown in Figure 1b.

The phase transition temperature can be effectively reduced by doping W^6+^ Nb^5+^ Cr^3+^ plasma or nanostructure VO_2_ [19]. In addition, the visible light transmittance of VO_2_ thin films is low (usually between 40~50%), resulting in the solar modulation ability being unsatisfactory [18]. 

Due to the above disadvantages of smart windows prepared by inorganic phase change material VO_2_ [20], polymer thermochromic smart windows have received extensive attention in recent years [19]. Compared with traditional inorganic thermochromic materials, organic polymer thermochromic materials possess low preparation costs and excellent sunlight regulation ability [10]. Moreover, most organic polymer materials are environmentally friendly and suitable to use in thermochromic smart windows [21].

**Figure 2:**
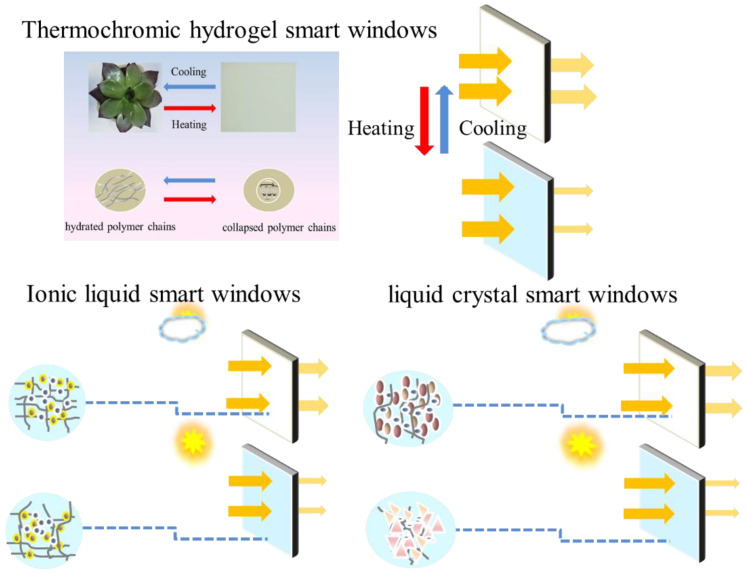
Illustration of thermochromic, Ionic liquid and liquid crystal smart windows.

In this review, hydrogels-based smart windows, including thermochromic ionic liquid and liquid crystal smart windows, are summarized and discussed (Figure 2). Firstly, the categories and strategies for the performance improvement of these three hydrogels-based smart windows are outlined. Secondly, the integration of the hydrogel with the multifunction devices was discussed. The last section summarizes the prospects of smart windows for future development and applications.

## 2. Thermochromic Hydrogel Smart Windows

The thermochromic smart windows use the characteristic that the light transmittance of thermochromic materials changes with temperature to dynamically adjust sunlight without consuming additional energy [22]. Another advantage of thermochromic smart windows is that it does not need an additional control system [23]. Thermally responsive organic materials, such as hydroxypropyl cellulose [24] and polyacrylamide [21], play an important role in thermochromic smart windows [25].

In addition, thermochromic functions can be combined with other functions, such as electrothermy-based active control and thermosensitive/electrochromic [26], for better light modulation and energy utilization.

### 2.1. PNIPAm-Based Thermochromic Materials Smart Windows

#### 2.1.1. PNIPAm Temperature-Responsive Hydrogel

A novel organic thermochromic material PNIPAm was used to replace the traditional inorganic phase change material VO_2_ [27]. With appropriate thickness, PNIPAm temperature-responsive hydrogel was completely transparent at room temperature, and the thickness of the thin layer reached 87.9%. Translucent at 40 °C [28], the thickness of the thin layer can reach 59.9%. Compared with the currently reported best VO_2_ thermochromic film (Δ*T*_sol(20–90 °C)_22.3%, *T*_lum(20 °C)_ 45.6% [13], and *T*_lum(90 °C)_ 40.0%) [29], they can provide both high modulation in the visible and infrared range, which lead overall to enhanced Δ*T*_sol(20–40 °C)_ of 20.4% or 25.5% for Δ*T*_sol(20–60 °C)_ [30].

**Figure 3:**
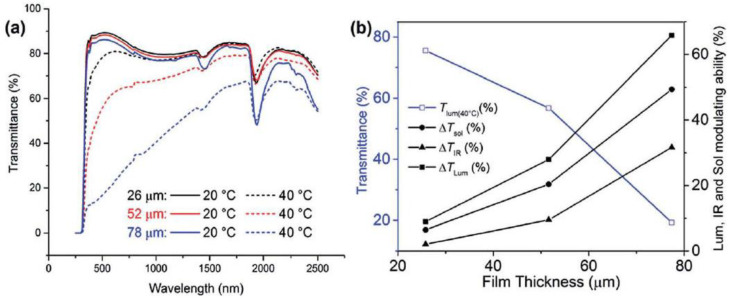
(**a**) Optical transmission spectra of three samples with different thicknesses at 20~40 °C. (**b**) The variation trend of Δ*T*_sol_, Δ*T*_lum_, Δ*T*_IR_ and Δ*T*_lum_ at 40 °C with different sample thicknesses [21,36].

According to the above discussion, the application of PNIPAm hydrogel film in smart windows is mainly related to the thickness of hydrogel [31]. The thickness effect of “thin” hydrogels with thickness less than 100 μm was further studied. When the thickness increases from 26 μm to 78 μm and the temperature decreases from 40 °C to 20 °C [32], the water absorption strength at 1930 nm and 1430 nm increases significantly (Figure 3a). It can be seen from Table 1 that the *T*_lum(20 °C)_ of the three samples remained unchanged [7], while the *T*_lum(40 °C)_ decreased monotonically from 80% (26 μm) to 20% (78 μm) [33]. While the 78 μm thick hydrogel showed impressive Δ*T*_sol_ values of nearly 50%, its low *T*_lum(40 °C)_ of less than 20% is less satisfactory for an ideal smart window [34]. *T*_lum_ at high temperatures and Δ*T*_sol_ are also suitable for hydrogels of 26 μm thickness [31]. At temperatures above lower critical solution temperature (LCST), extremely low thickness accompanied by almost constant brightness transmittance and poor solar regulation ability occur [35]. Averaged *T*_lum(40 °C)_ decreases with thickness, Δ*T*_lum_, Δ*T*_IR_ and Δ*T*_sol_ increases with the thickness (Figure 3b) [36].

This high-performance temperature-responsive hydrogel can facilitate the development of thermochromic smart windows based on organic materials [35].

#### 2.1.2. PNIPAm Thermally Responsive Liquid Hydrogel

Conventional smart windows can only adjust solar energy transmission [33]. Researchers extracted the hydrogel-derived liquid in glass [37] and developed the high thermal energy storage thermoresponsive smart window (HTEST smart windows) [15]. Excellent thermal response optical properties, coupled with high liquid specific heat capacity, enable HTEST smart windows to have excellent energy-saving performance. Simulation results show that, compared with ordinary glass in Singapore [38], the HTEST windows can reduce the energy consumption of heating, ventilation and air conditioning by 44.6%. In the outdoor demonstration, HTEST smart windows showed good energy-saving performance during the summer day [39].

**Figure 4:**
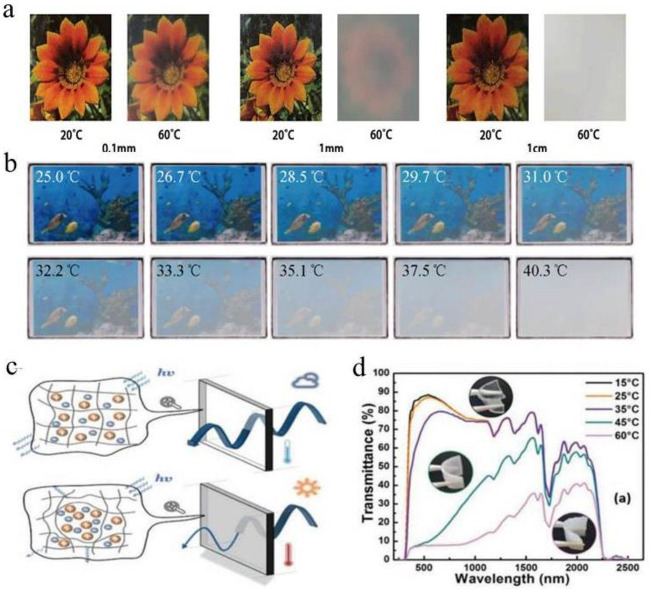
(**a**) Optical photographs of 0.1 mm, 1 mm and 1 cm samples at 20 °C and 60 °C. (**b**) Schematic diagram of thermochromic optical transmittance as a function of temperature. (**c**) Schematic diagram of the change of thermochromic light transmittance. The reduced transparency of the ionic liquid above the LCST is due to light scattering at the interface between the ionic liquid and the polymer chain. (**d**) Transmission spectra of the UV-Vis-NIR regions at different temperatures of 15 to 60 °C [5,42].

Optical photos of samples of different thicknesses at 20 °C and 60 °C. The optical image is consistent with spectrum: at low temperatures, all samples are transparent light transmittance free from thickness [13]. Moreover, when the temperature is higher than LCST [36], transmittance of 0.1 mm sample [40] does not change significantly. In contrast, the 1 mm sample becomes translucent [41], the 1 cm sample becomes opaque, and flowers under the 1 cm sample become invisible (Figure 4a) [42]. Therefore, thermal response optical characteristics of thermoresponsive liquid (TRL) can be adjusted by changing temperature as well as thickness of TRL [43] significant difference in optical transmittance (Figure 4b). By adjusting the hydrophilic and hydrophobic balance of thermochromic hydrogels through copolymerization, the phase transition process can be flattened, while the transmittance near the phase transition temperature changes slowly with the change of ambient temperature (Figure 4c). Transmittance spectra of an ion gel at different temperatures of 15~60 °C. The spectral range of 300~2200 nm is affected, with temperature increase of ion gel. when the ion gels were heated above LCST, up to 80% luminous modulation (Δ*T*_lum_) was to be achieved by changing the observable color to white (Figure 4d), as reported by Lee et al.

Compared with traditional energy-saving smart windows [44], which require expensive equipment, the thermally responsive liquid structure is easy to manufacture, has good uniformity and expansibility and has the function of sound insulation, which opens a new path for energy-saving buildings and greenhouses [45].

#### 2.1.3. PNIPAm/Antimony-Doped Tin Oxide (ATO) Nanocomposite Hydrogel

**Figure 5:**
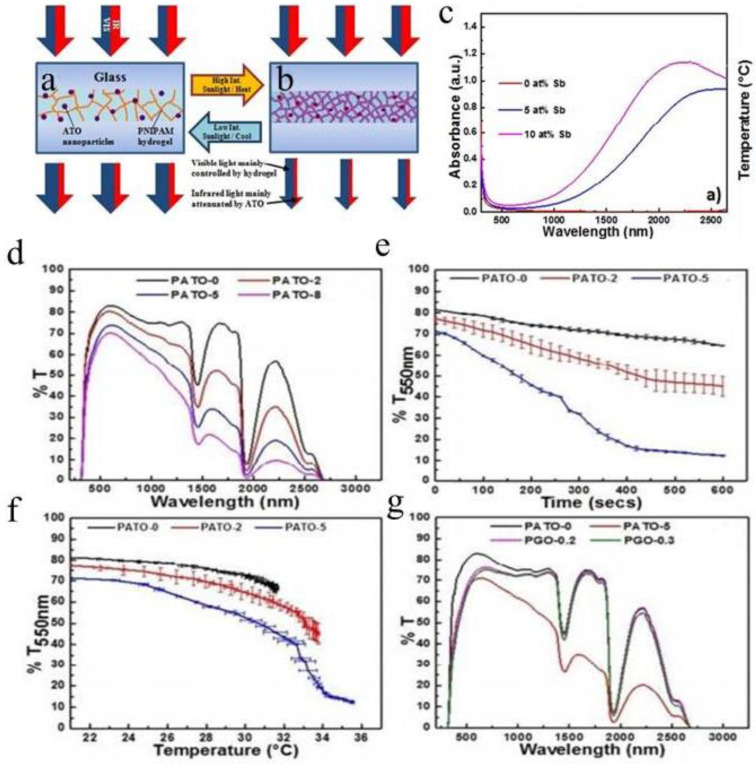
Optical sensitive smart windows (**a**) transparent state and (**b**) translucent state structure diagram. (**c**) UV-Vis-NIR absorption spectra of 0.025 wt% SnO_2_ dispersed in perchloroethylene solvents with Sb doping of 0, 5 and 10 at% [49]. (**d**) UV-Vis-NIR spectra of nanocomposite hydrogel films with different filling contents at 25 °C. (**e**) Single-wavelength transmittance rate (*T*_550_%) nanocomposite hydrogel as a function of € time and (**f**) temperature from solar irradiation at 100 mW cm^−2^ for 10 min. The figure above shows the optical properties of the 180 nm hydrogel film incorporated with various content of 10 at% Sb doped ATO. (**g**) UV-Vis-NIR spectra of PATO Compared with different levels of PGO at 25 °C [50].

Photothermal and temperature-sensitive nanocomposite composed of hybrid poly PNIPAm hydrogel and antimony-doped tin oxide (ATO) were reported by the Li group [44]. In this photothermal system, near-infrared (NIR) absorption of ATO can be used as an optical switch to induce hydrogel induced by nano heater [46]. This new type of passive smart windows has excellent near-infrared shielding performance. The photothermal activation switch mechanism enhances the thermal response speed and solar modulation capability. [47] ATO doped with 0, 5, 10 and 15 at% Sb separately in PNIPAm was studied, it was found that PNIPAm/ATO nanocomposites could be photothermal activated [48]. The PNIPAm /ATO doped with 10 at% Sb showed the best thermal response speed and solar modulation ability. Different film thicknesses and ATO content will affect the response rate and solar modulation ability. By increasing the content of Sb dopant in SnO_2_ to 10 at%, the concentration of free electrons increases, leading to the improvement of near-infrared absorption efficiency. This means ATO can convert absorbed solar radiation into heat, also known as the photothermal effect. For structural stability, the performance stability of such a photothermal system [49] can be proved by 15 consecutive periods of irradiation under solar intensity (Figure 5c).

When PNIPAm/ATO composite hydrogels (PATO) are exposed to sunlight irradiation (Figure 5a,b) [17], dimming control can be facilitated and accelerated by plasmonic heating of ATO under glaring sunlight even if the temperature outdoor is far below the transition point of PNIPAm hydrogel [45].

As the content of ATO in PATO increases, the absorption of the plasma increases, resulting in a decrease in transmission in the NIR region (Figure 5d). With the increase of ATO content, a significant increase in the response rate is observed in addition to the lower visible light transmittance in the transparent state (Figure 5e). The optical effects of different ATO concentrations on the PATO are further observed in the transmittance temperature profile (Figure 5f) [45]. Use ATO as an alternative to graphene oxide (GO), as a nano heater. Their performance is compared in (Figure 5g). Unlike the PATO showing the NIR shielding effect, the PGO shows only a decreased visible transmittance as the GO content increases, while the spectrum in the NIR region remains the same as the PNIPAm [17].

In the background of the smart windows, NIR absorption is an important feature of indoor temperature regulation. Thus, not only does it sacrifice the photothermal rate of visible light, but PGO also fails to achieve significant NIR shielding and insulation. So, in this respect, PATO will be more capable than PGO. [50].

In addition to the dimmer-controlled transparency modulation, the addition of ATO also helps in NIR shielding to reduce burden of cooling and air conditioning in tropical climates [44]. A series of parameters including ATO doping, composite hydrogel thickness and nanoparticle content were studied and optimized. We feel that the experimental results of the study will provide further research direction for development of ideal passive smart glass [51].

We believe that this new generation of autonomous passive smart windows can be used to adapt to solar modulation.

#### 2.1.4. PNIPAm/VO_2_ Nanocomposite Hydrogel

Although vanadium dioxide is a hot spot in the areas of color-changed materials [31], it has low solar energy modulation (Δ*T*_sol_) and low luminous transmittance (*T*_lum_) and high phase change temperature [50].

**Figure 6:**
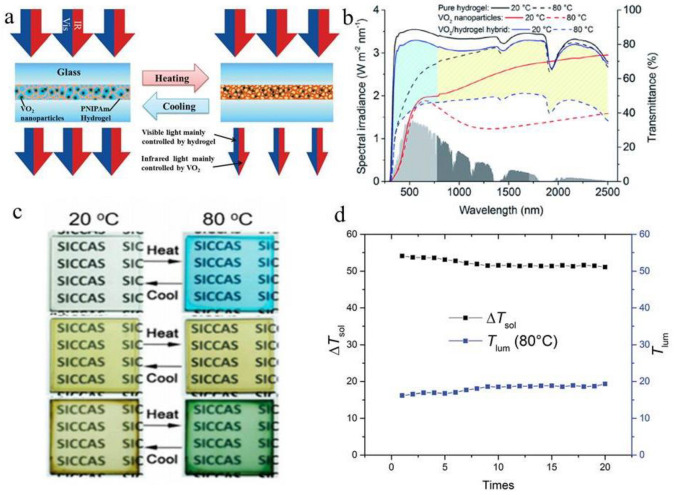
(**a**) Sunlight regulation mechanism of VO_2_/ hydrogel composites. (**b**) UV-Vis spectra of pure hydrogels, pure VO_2_ and VO_2_/ hydrogel complexes [51]. (**c**) Films Photographs based on the pure IL-Ni-Cl complex (above row), VO_2_ nanoparticles (intermediate row) and VO_2_/IL-Ni-Cl composite (bottom row) at 20 °C (left column) and 80 °C (right column), respectively. The IL-Ni-Cl complex and VO_2_/IL-Ni-Cl composite have significant temperature-responsive color changes. (**d**) Durability test of composite hydrogels with the thickness of 100 μm from 20 °C to 80 °C. [52].

Traditional hydrogel smart windows have higher modulation efficiency in visible light wavelengths, while light modulation efficiency in near-infrared wavelengths is lower. To improve the solar modulation efficiency of smart hydrogel windows, researchers combined organic and inorganic thermochromic materials to prepare organic/inorganic composite thermochromic hydrogels [52]. Hydrophilic and hydrophobic phase transition of PNIPAm at 30 °C mainly controls the light emission modulation [53], while the monoclinic phase transition of VO_2_ nanoparticles at 68 °C mainly controls infrared modulation [54]. The formation mechanism of the layered VO_2_/hydrogel hybrid film is shown in Figure 6a. In 2015, Long Yi’s research group dispersed VO_2_ in PNIPAm hydrogel and prepared an organic/inorganic hybrid smart window with high visible light transmittance when the temperature was lower than phase transition temperature [51]. Organic/inorganic hybrid smart windows also have a high solar modulation efficiency, which is more than twice that of the traditional VO_2_ thermochromic smart windows [55]. Figure 6b shows the transmittance of the hybrid smart window before and after the phase change.

In another work, the Jin׳s team combined VO_2_ nanoparticles with thermochromic ionic liquids, which usually increase the absorbance at 650–750 nm when heated, instead of increasing the absorbance over the entire visible light range like hydrogels. The VO_2_/IL-Ni-Cl composite has excellent optical regulatory properties (Δ*T*_sol_ of 26.5%) and maintains good transparency (*T*_lum_ of 50%). More interestingly, these films present significant color changes from brown (20 °C) to green (80 °C), which are considered synergistic of color variations in pure VO_2_ and pure IL-Ni-Cl films (Figure 6c). The proposed method provides an alternative for simultaneously improving the unfavorable brown-yellow color of VO_2_ and achieving good solar modulation. Then they were applied to a ligand exchange thermochromic system based on cobalt (II) and nickel (II) (Co-based ligand exchange thermochromic system and Ni-based ligand exchange thermochromic system) and obtained similar results [51].

The VO_2_/hydrogel composite maintains good reversibility during the durability test. *T*_lum_ and Δ*T*_sol_ are relatively stable at 80 °C, and there is almost no change in 20 cycles of measurement. This shows that in actual smart window applications, as long as good sealing is achieved and moisture evaporation is prevented, the performance stability should be reliable (Figure 6d) [55].

The composite material proposed by the researchers has unprecedented excellent thermochromic properties, opening a new research direction for the field of thermochromic smart windows in the future.

#### 2.1.5. Graphene Oxide (GO)/PNIPAm Nanocomposite Hydrogel

**Figure 7:**
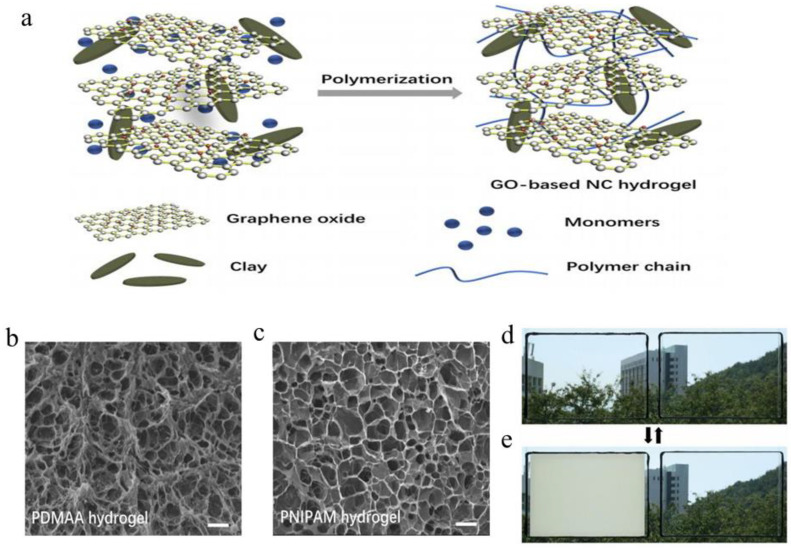
(**a**) Schematic illustration of GO-based NC hydrogel components. (**b**) SEM image of GO-based NC PDMAA hydrogel. (**c**) SEM image of GO-based NC PNIPAm hydrogel. The scale bars are 10 μm. 30 °C Changes of light transmittance between PNIPAm/GO composite hydrogel (left) and PNIPAm hydrogel (right) (**d**) before thermal radiation; (**e**) after thermal radiation [59].

Regarding the bilayer structure of the hydrogel, mechanical properties of poly (*N*,*N*-dimethylacetamide) PDMAA and PNIPAm are studied respectively. Figure 7a illustrates the composition and polymerization process of a nanocomposite (NC) hydrogel based on graphene oxide (GO).

The monomers (DMAA and NIPAM), dissolved in GO dispersion separately, cannot only react with a crosslinking agent to form a covalent bond but also has a noncovalent bond with the clay due to rich amide groups. Thus, these monomers tend to polymerize next to the clay plate to form noncovalent bonds, as a result, enhancing the mechanical properties of the hydrogel [55]. There are active groups on GO sheets such as –COOH and –OH to easily disperse in water, which facilitates the consistency between polymers and GO sheets in the hydrogel system. The internal morphology of the individual PDMAA and PNIPAm hydrogels was shown by SEM (Figure 7b,c) [55]. Both hydrogel networks have a nearly uniform aperture, while the GO is well dispersed in the hydrogel without any aggregation, suggesting that the gel isotropic structure has uniform mechanical properties [56].

In 2014 [57], Chou prepared GO/PNIPAm thermochromic material using GO dispersion as photothermal conversion material. Subsequent tests showed that at 30 °C (<LCST), PNIPAm with GO dispersion could still undergo a phase transition in a relatively short period [56]; however, pure PNIPAm hydrogel smart windows could not complete phase transition (Figure 7d,e). The addition of GO is beneficial to improve the practical application of PNIPAm hydrogel in the field of smart windows [58].

Recent strategies of the fabrication of thermochromic hydrogel smart windows, including their name, function, thermochromic properties and characteristics are summarized in Table 2. Noncovalent interactions responding to temperatures, such as precise intermolecular hydrogen bonding control between polymer chains and water molecules, could optimize the performance of hydrogel smart windows. The pure hydrogel smart windows system is easy to fabricate with excellent solar modulating ability. With the addition of IR regulating/blocking materials, the solar modulating ability of thermochromic hydrogel smart windows was further enhanced. By adding photothermal nanoparticles (ATO, GO), the response speed of smart windows was further improved. At the same time, thermochromic host–guest interactions of hybrid hydrogels were fabricated to improve mechanical stability.

### 2.2. Hydroxypropyl Cellulose (HPC) Based Thermochromic Materials Smart Windows

Thermochromic materials are the most cost-effective smart windows. Compared with inorganic VO_2_ materials, organic smart materials have higher solar energy modulation (Δ*T*_sol_) and luminous transmittance (*T*_lum_) [60].

In 1998, Watanabe et al. dispersed hydroxypropyl cellulose in 5% sodium chloride aqueous solution [59], obtained an intelligent window with the area of 1 m^2^ and found that its cyclic stability and strong optical properties were superior [59]. In 2001, Schneider and See both blended hydroxypropyl cellulose with hydroxyethyl cellulose and dispersed it to gum [44,60], which not only inhibited water volatilization but also improved optical properties [44].

In 2016, Long reported green and new organic thermochromic materials hydroxypropyl cellulose [61]. With addition of sodium chloride (NaCl) from 0.5% (wt.%) to 5% (wt.%), (lower critical solution temperature) LCST decreased from 42 °C to 30 °C [21]. From morphology changes of LCST and freeze-dried samples, it can be seen that the phase transformation of hydroxypropyl cellulose hydrogels is caused by the different solubility of polymers in water [62]. The fine-tuned recipe could afford an outstanding solar modulating ability (Δ*T*_sol_) of 25.7% and a high averaged *T*_lum_ of 67.4% with an LCST of 38 °C [63].

**Figure 8:**
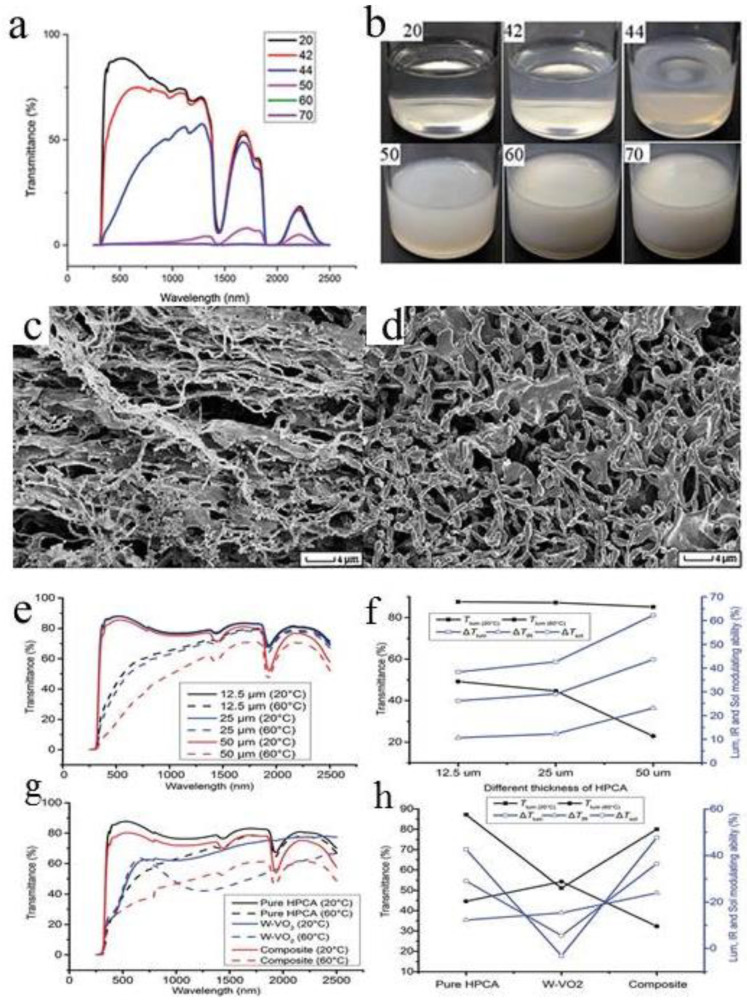
(**a**) Optical transmission spectrum of 0.35 mm HPC sample at 25–50 °C. (**b**) Pictures of HPC at different temperatures. SEM images of freeze-dried HPC microgel at (**c**) 20 °C and (**d**) 80 °C, respectively [68]. (**e**) Transmission spectra of 12.5, 25 and 50 μm thick HPC microgel samples; (**f**) The effect of thickness on the HPC samples Δ*T*_sol_, Δ*T*_lum_, Δ*T*_IR_ and *T*_lum_ (20 and 60 °C); (**g**) Transmission spectra of 25 μm HPC, W-VO_2_, W-VO_2_ and HPC microgel samples [62] (**h**) Δ*T*_sol_, Δ*T*_lum_, Δ*T*_IR_ and *T*_lum_ (20 and 60 °C) of 25 μm HPC, W-VO_2_ and W-VO_2_ with HPC microgel samples [70].

When temperature is heated from 20 °C to 44 °C, the visible light transmittance of HPC decreases significantly, while the change of IR [64] can be ignored (Figure 8a). This reflects change of solution from transparent at 20 °C to translucent at 42 °C (Figure 8b). It is worth noting that the transmissivity decreases around 1430 nm and 1930 nm at both high and low temperatures, as a result of absorption of water at these two wavelengths [65]. When temperature rises to 50 °C, the visible and infrared transmittance of the solution decreases significantly, and the opacity of the solution also increases [26]. When temperature rises to 60 °C and 70 °C, it becomes completely opaque while all transmissions at 250~2500 nm are blocked. Transparency is restored. within 1 min after the heater is removed.

The difference of the HPC structure at 20 °C and 80 °C is shown in Figure 8c. At room temperature, the elongation and homogeneity of polymer particles are stronger. However, at 80 °C, the length of polymer fibers decreases sharply to about 4–5 μm, with a large shrinkage pore size (Figure 8d).

Thermosensitive microgels were directly synthesized from hydroxypropyl cellulose (HPC) and acrylic acid (AA) in pure water [66]. The phase transition temperature of the inorganic/organic hybrid thermochromic material is around 45 °C, which is obtained by dispersing the W-doped VO_2_ in the HPC microgel. The composite microgel has high transparency at room temperature with a thickness of 25 μm, *T*_lum_ = 80% [67]. Compared with pure VO_2_ inorganic materials and previously reported thermochromic composites, it has 36% excellent solar light (*T*_sol_) modulation ability, 56% average *T*_lum and_ a phase transition temperature of 50 °C, which makes it suitable for intelligent window applications [68].

Optical transmission spectrum of HPC microgel samples with thicknesses of 12.5 μm, 25 μm and 50 μm respectively (Figure 8e). The *T*_lum_ of 20 °C of the HPC membrane dropped from 87.5% to 85.1% at 20 °C; however, at 60 °C, The *T*_lum_ of HPC membrane decreased from 49.2% to 22.9%. Increasing from 12.5 μm to 25 μm Δ*T*_sol_, Δ*T*_lum_ and Δ*T*_IR_ increased slightly and dramatically from 25 μm to 50 μm (Figure 8f). [69] Although the 50 μm thick HPC microgel has a ΔT_sol_ value of 44%, its T_lum_ (60 °C) of below 25% is not fit as an ideal smart window [61]. According to the figure above, the pure HPC microgel of 25 μm thickness is best suitable for smart windows as compared to 12.5 μm and 50 μm. The transmission spectra of 25 μm thickness samples of HPC, W-VO_2_ and W-VO_2_ HPC composite microgel (250~2500 nm) samples at 20 °C and 60 °C (Figure 8g) [68]. Pure HPC films have the highest solar transmittance at 20 °C, with a *T*_lum_ value of 87%. The pure HPC microgel Δ*T*_lum_ is 43% and the sample becomes opaque when the temperature rises to 60 °C. There is still a large transmission ratio for pure W-VO_2_ in IR region, *T*_IR_ is up to 15% compared to a Δ*T*_sol_ of 5%, which is a better smart IR blocker in the HPC microgel. Although the added W-VO_2_ transmittance decreased at 20 to 60 °C, and the HPC film added to W-VO_2_, Δ*T*_IR_ increased twofold from 12–24% compared with that of the pure HPC film (Figure 8h), while Δ*T*_lum_ also rose from 42.6% to 47.5%, resulting in a 25.5% increase in Δ*T*_sol_. Therefore, compared with pure HPC and W-VO_2_ films, the new hybrid film has greater modulation capability in visible and infrared wavelet bands [69]. At the same time, at room temperature, a very high Δ*T*_sol_ can be obtained with a visible light transmittance of only 7.2% [70]. This is the first device that can effectively block visible and infrared light at the right phase transition temperature.

### 2.3. Brief Conclusion

Mainly introduces the type of thermochromic hydrogel materials, the preparation method and performance of smart windows. Pure hydrogel smart windows are simple to make and have good solar modulation ability. With the addition of infrared regulation materials, the adjustment ability of solar thermal chromic hydrogel smart windows is further enhanced, with a faster response speed. In addition, the recombination of thermochromic materials and electrochromic materials can better improve the performance and mechanical stability of the hydrogel. Finally, to commercialize smart windows, the materials and technology are durable, cheap and easy to handle.

## 3. Ionic Liquid Smart Windows

Ionic liquids (IL) including polymer ionic liquids (PIL) are a rapidly developed and extensive field of research due to their unique properties such as nonvolatile [71], high ion conductivity, electrochemical stability and solvation potential [72,73].

### 3.1. Organic and Inorganic Complex Ionic Liquid Smart Windows

The ionic liquid-based composite film, a mixture of transition metal compounds and ionic liquid, [74,75,76,77] has much attention as a promising energy-saving smart windows candidate due to its ability to regulate solar radiation through absorption. [78,79,80,81,82,83,84,85,86].

Current research in this field is mainly focused on thermochromic ionic liquids, [87,88] whose property is based on an octahedral–tetrahedral configuration change of transition metal complexes assisted by interaction with donor solvent molecules [34,89].

**Figure 9:**
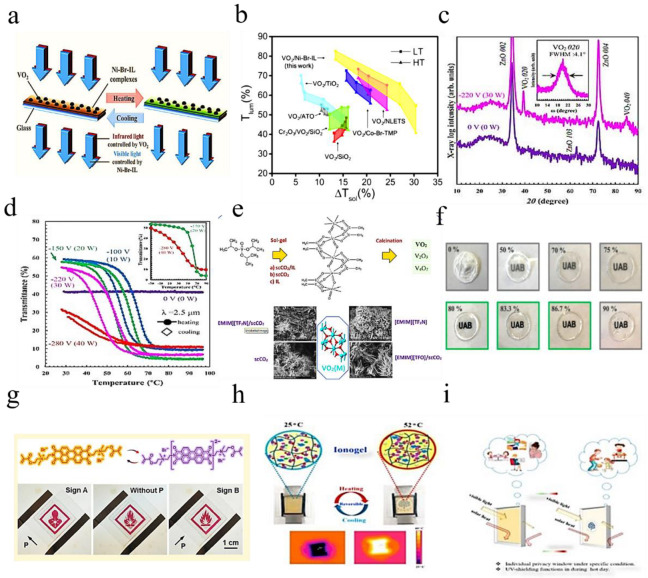
(**a**) The thermochromic mechanism of VO_2_-based thermochromic film. (**b**) Luminous transmittance (*T*_lum_) and solar regulation efficiency (Δ*T*_sol_) of different VO_2_-based composite films [71]. (**c**) XRD patterns of VO_2_/ZnO/glass samples prepared at substrate bias powers of 0 and 30 W, respectively [90]. (**d**) The optical transmittance changes of all samples at 2.5 μm. (**e**) Vanadium oxide VO_2_ (M) synthesis was investigated using supercritical CO_2_ (scCO_2_), imidazolium ionic liquids (ILs) and their biphasic combination. (**f**) IG prepared with different weight ratios of BMIM TFSI shows the change in transparency after adding BMIM TFSI [93]. (**g**) Adjust the chemical structure of PDI-MA and PDI-MA dianion through reversible redox reactions. (**h**) The image of the ion gel-based device 1 (with water) corresponds to the temperature dependence of the transmittance change from 25 °C to 52 °C. (**i**) The working principle of smart windows is based on ionic gel materials under specific conditions [86].

A new thermochromic ionic liquid (nickel-bromide-ionic liquid (or Ni-Br-IL)) combined with VO_2_ nanoparticles enhances optical performance [71]. This enhanced performance will benefit applications based on VO_2_-based smart windows. The thermochromic mechanism of the VO_2_/Ni-Br-IL composite film is shown in Figure 9a and b, showing the comparison of VO_2_-based composite films with optimized optical properties reported in recent years. According to reports, some materials such as TiO_2_ and SiO_2_ can be used as anti-reflection layers to effectively improve the optical properties of VO_2_-based composite films.

In Figure 9c, the c-axis oriented growth of the ZnO film on the glass is observed, with strong diffraction from ZnO 002 and 004. Without bias (0 W), no significant peaks from VO_2_ were observed. [90] Figure 9d shows the temperature dependence of transmittance at a wavelength of 2.5 μm. The researchers recognized a behavior similar to the R-*T* characteristic, in which the transition temperature shifts to the lower side as the bias power increases.

The synthesis of vanadium oxide VO_2_(M) was investigated using supercritical CO_2_ (scCO_2_), imidazolium ionic liquids (ILs) and their biphasic combination (Figure 9e). VTIP was used as the vanadium precursor while examining [EMIM] ionic liquids based on anions of [TF_2_N]/[TFO]/[AcO]/[HSO_4_]. [91] Using scCO_2_/HOAC or using only [EMIM][TF_2_N] IL resulted in a gelation yield of up to 80%, indicating the role of scCO_2_ in the gelation process. It is found that the sol-gel temperature has a greater influence on the gelation yield than the pressure.

The IG membranes presented in Figure 9f were studied. They contain different cation–anion compositions, ionic liquid loading and dopants [73]. By loading different amounts of IL into the P(VDF-co-HFP) polymer matrix and comparing their optical, electrical and mechanical properties, the influence of the IL content (weight percentage) in IG was evaluated. The combination of BMIM^+^ cation and TFSI^−^ anion were used as the model IL. According to the IL load (Figure 9g), the obtained IG film showed obvious differences, which was confirmed by spectral analysis [92]. At present, 3-chloro-*N*-isopropylacetamide containing thermally responsive poly [VNIm][TFSI] based electroactive substances were synthesized for use in multi-responsive devices [93]. Ion gel, consisting of MBV [TFSI], ferrocene and poly [VNIm][TFSI], is used to construct single layer (all-in-one) devices (with and without water). When the temperature drops below the UCST of poly [VNIm][TFSI], the intermolecular hydrogen bond and hydrophobic anion (TFSI) produce a heterogeneous state (opaque), so the device 1 becomes opaque, as shown in Figure 9h [77,92]. This thermally responsive material is very useful for smart windows under certain conditions, as shown in Figure 9i [86].

### 3.2. Multi-Functional Organic Ionic Liquid Smart Windows

**Figure 10:**
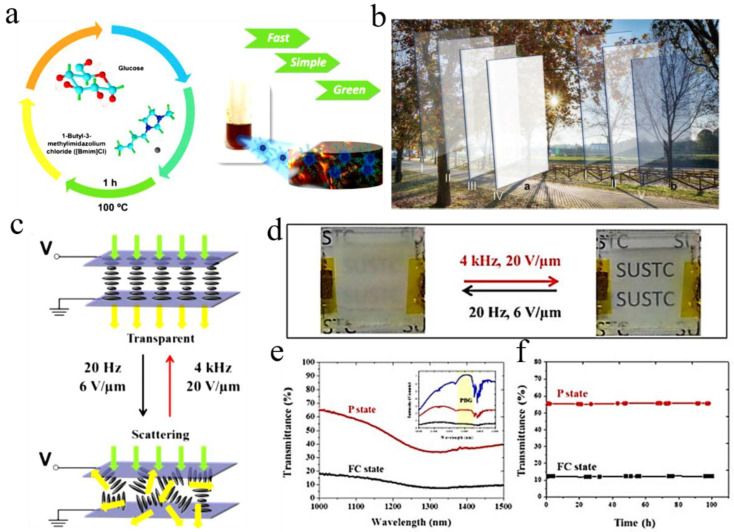

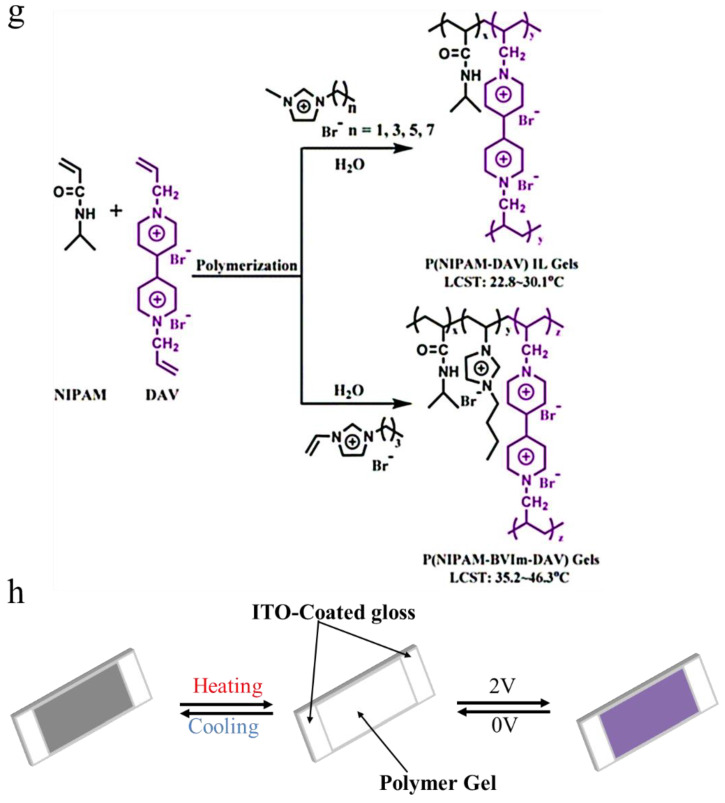
(**a**) Schematic representation of the nanofluid synthesis, 1-butyl-3-methylimidazolium chloride as both reaction medium and anhydrous glucose as starting material and functionalization molecule [99]. (**b**) Schematic representation of the operation of a window glass incorporating the surface plasmon resonance effect (SPRE) enhanced thermotropic device (**a**) the integrated electrochromic device/thermotropic device system (**b**) Outside view at 25 °C (I), 36 °C (II), 50 °C (III), 60 °C (IV) [89]. (**c**) The smart window is driven by dual-frequency modulation and is transparent at a high-frequency electric field (4 kHz) and scattered at a low-frequency electric field (20 Hz). (**d**) Images of CILC smart window in transparent P state and scattering FC state [101]. (**e**) (Inset) Transmittance intensity of reference light (blue curve), P state (red curve) and FC state (black curve). Wavelength-dependent transmittance of FC texture (black curve) and P texture (red curve). (**f**) Transmittance stability of smart window versus time of which the textures are in FC state (black curve) and P (red curve). (**g**) General synthetic routes to P(NIPAM–BVIm–DAV) and P(NIPAM–DAV) IL gels. (**h**) The principle and mechanism of thermochromic and electrochromic devices based on the prepared polymer gel [98,102].

The design of new materials and new technologies is essential to the development of smart windows in next-generation energy-efficient buildings [94,95,96,97]. It is shown that the glucose derivative with 1-butyl-3-methylimidazolium chloride can form a self-supporting, water-soluble, viscous, reusable nanofluid, with self-improved conductivity, thermal degeneration around 30–40 °C and the ability to block ultraviolet rays (Figure 10a) [83]. In the hot summer months of 36 °C, sun-actuated thermotropic (TT) devices containing a 95% nanofluid aqueous solution exhibit a transmittance variation (Δ*T*) of 9% at 550/1000 nm, amplified to 47% through a surface plasmon resonance effect [98]. The integrated self-healing system is capable of independent sun-actuated thermotropic (TT) and voltage-actuated electrochromic (EC) operation (Figure 10b) [94].

Then a bistable smart window with mixture of chiral dopants and chiral ionic liquid (CIL) was developed [83]. The smart windows are driven by dual-frequency modulation and are transparent at a high-frequency electric field (4 kHz) and scattered at a low-frequency electric field (20 Hz) (Figure 10c) [99].

Compared with the PSCT smart windows, the switching between the scattering and transparent states can be dynamically controlled by dual-frequency modulation, without the use of high-field H states [80]. During the conversion process, the scattering state was turned on by a low-frequency electric field (20 Hz, 6 V/μm) and the transparent state was triggered by a high-frequency electric field (4 kHz, 20 V/μm). The uniformity is quite good (Figure 10d) [34,88].

The red and black curves represent the transmittance spectra of the P state and FC state, respectively. The blue curve in the illustration indicates the intensity of reference light. In both P state and FC state, a transmittance of the infrared [89]. The reference light of the CIL-doped cholesteric liquid crystal (CILC) smart windows is reduced compared to the illumination intensity, mainly due to blocking of the liquid crystal (LC) film [98]. A notable decrease in light transmittance can be observed in the range of 1250∼1350 nm, at P state, indicating the Bragg reflection band is formed (Figure 10e).

At P state (red curve) and focal conic (FC) state (black curve) plots the temporal fluctuation in transmittance of the CILC smart windows. It shows a very small fluctuation of transmittance after the removal of voltage (Figure 10f) [100].

Synthetic route of the polymer gel studied. The polymer gels were synthesized by co-polymerization of *N*-isopropyl-acrylamide (NIPAM) and Diallyl-viologen (DAV), with (or without) an IL monomer, 3-butyl-1-vinyl-imidazolium bromide([BVIm][Br]) (Figure 10g) [81]. The principle and mechanism of thermochromic and electrochromic devices based on the prepared polymer gel (Figure 10h).

### 3.3. Brief Conclusion

Table 3 summarizes the latest preparation strategies for ionic liquid smart windows, including names, functions, thermochromic properties and features. The unique properties of the ionic liquid and polymer combined with the thermal response can be further developed. Here, we present the potential applications of customizable ILs/PILs in the emerging field of solar-light modulation.

## 4. Liquid Crystal Smart Windows

Liquid crystal (LCS) represents a new material between traditional liquid and crystalline solid that can flow like a liquid or in the form of crystal [103]. Most liquid crystals are made up of organic compounds. They have the properties of optical, electrical and mechanical anisotropy. These properties are responsive to various stimuli, such as temperature, electric field, light, which makes LCS valuable in a wide range of applications [104].

### 4.1. Liquid Crystals Based Flexible Smart Windows

The optical properties of LCS are highly dependent on the orientation of the isotropic molecule. The thermochromic performance of LCS is achieved by adjusting in response to temperature stimuli [20,22,53,105,106,107,108]. In the smart window of the sandwich structure, LCS molecules can be adjusted in multiple directions between two glass slides. Three typical (uniform) directions of the slide [16,35,109].

**Figure 11:**
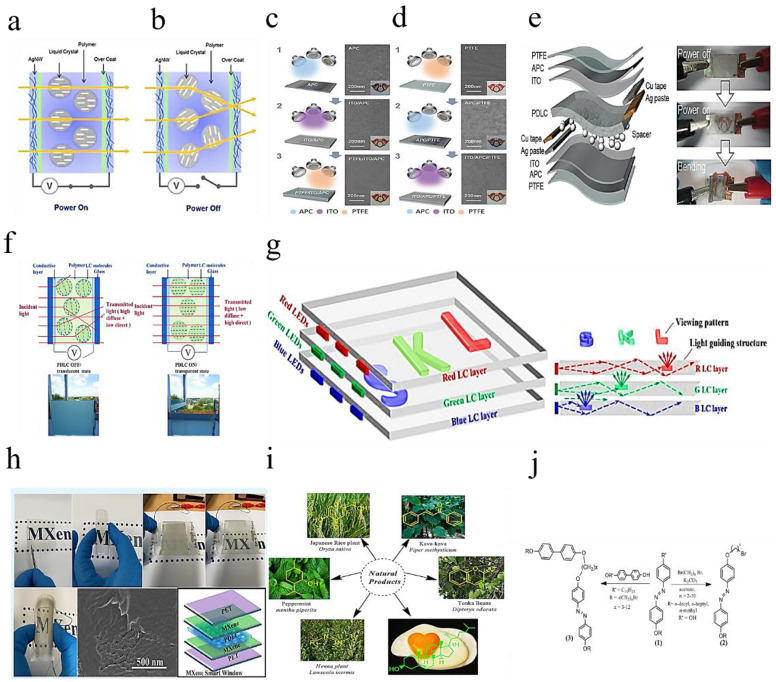
(**a**) Transparent and (**b**) opaque states are caused by applying an external voltage between the Ag NW electrodes [110]. (**c**) PTFE/ITO/APC (**d**) ITO/APC/PTFE hybrid electrode on CPI substrate at room temperature. (**e**) Polymer dispersed liquid crystal (PDLC) switchable smart windows for “opaque/translucent” (OFF) and “transparent” (ON) states [109]. (**f**) Schematic of a design example to present (**g**) multi-layered LC smart windows with the colored logo of “SKL” [111]. (**h**) Ti_3_C_2_Tx MXene has excellent performance as a two-dimensional material [112]. (**i**)Azo incorporated natural products for LC derivatives. (**j**) Azo-benzene liquid crystal derivatives [115].

Figure 11a shows a schematic diagram of the device structure and operation of the Ag NW-based flexible PLDC windows manufactured by the RTR slot die coating system [110]. The polymer–liquid crystal composite is sandwiched between two RTR slit die-coated Ag NW electrodes. If the refractive index of the liquid crystal along the alignment direction matches that of the polymer, the PDLC composite between the Ag NW electrodes allows light to pass in a transparent “on” state (Figure 11a) [109]. When the external voltage is removed, the liquid crystal is oriented in a random direction and scattering incident light. This opaque state is “off”, as shown in Figure 11b. Therefore, high-quality transparent electrodes with low sheet resistance, high light transmittance and good mechanical flexibility are very important in the operation of flexible PDLC windows [111].

To investigate the stacking sequence effect of the multilayer, PTFE (40 nm)/ITO (35 nm)/APC (10 nm)/CPI and ITO (35 nm)/APC (10 nm)/bottom PTFE (40 nm)/CPI samples were prepared, as shown in Figure 11c–e [112]. Considering the large-area smart windows used in buildings and automobiles, we adopted a mature sputtering method, using PTFE polymer targets, APC metal targets and ITO ceramic targets at room temperature. Surface field emission scanning electron microscopy (FESEM) images on the right side of the figure show the surface morphology of each sputtering layer. The ITO and APC layers of the DC sputtering all show a typical oxide and metal surface morphology, regardless of the sputter order [110].

The sputtered PTFE layer also exhibits featureless and smooth surface morphology similar to typical polymer films. Insets show each sputtering layer [113].

Figure 11f shows a schematic of the design example presenting the “SKL” color logo, where one of the ITO substrates of each LC unit is patterned as “S”, “K” and “L”. Then the patterned substrate is then assembled with another non-patterned ITO substrate to form a complete single-layer liquid crystal unit.

Figure 11g shows the light propagation process in the multilayer LC smart windows. The incident light emitted by the LED light bar enters the corresponding liquid crystal cell and propagates in the liquid crystal cell according to the law of total internal reflection.

Figure 11h Among the various materials available for transparent and flexible devices, MXenes is attracting attention as a new candidate material in this category [114]. Ti_3_C_2_Tx MXene has excellent properties as a 2D material, making it a potential material with many applications in different fields. Due to its high conductivity, it can be used for transparent conductive electrodes (TCE).

Azo-conjugated natural product derivatives are shown in Figure 10i. Azo is a well-known molecule with LC properties. It is a dye with a chromophore (^−^*N*=*N*^−^). Azobenzene is a reactive precursor in LC chemistry because the presence of the azo scaffold (^−^*N*=*N*^−^) has excellent absorption in the visible light region and has light and thermal stability. Azobenzene can be synthesized by diazotization of amine or phenol and diazotized amine. Figure 11j Liquid crystal derivatives of azobenzene [115].

### 4.2. Multi-Functional Liquid Crystal Smart Windows

Multi-functional liquid crystal smart windows have been commercialized, and there are some studies in the literature, such as cholesteric liquid crystal (CLC) units, polymer-stabilized liquid crystal (PSLC) and polymer dispersed liquid crystal (PDLC) [64,101,116,117].

**Figure 12:**
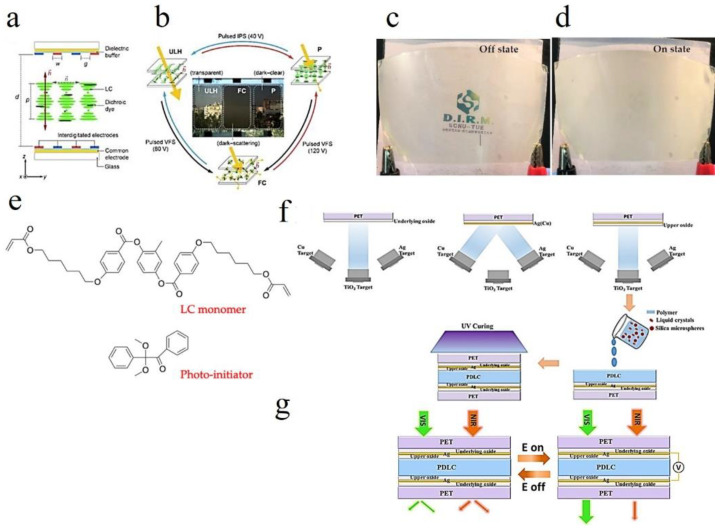
(**a**) Schematic depiction of six terminal cells containing dye-doped cholesteric liquid crystal (CLC) [105]. (**b**) Drive scheme and photographs of tristable CLC smart windows. (**c**) PSLC on PET substrate is off state [99]. (**d**) PSLC on PET substrate is on the state. (**e**) Molecular structure of chemicals used to manufacture a polymer-stabilized liquid crystal (PSLC) [85]. (**f**) fabrication process of the oxide/Ag/oxide electrode-based polymer dispersed liquid crystal (PDLC) device. (**g**) two optical modulation modes of the oxide/Ag/oxide electrode-based polymer dispersed liquid crystal (PDLC) devices [41].

The cholesteric liquid crystal (CLC) cell has interdigitated electrodes and a common electrode on each substrate (Figure 12a), thus forming an architecture that can employ various drive schemes [82]. Two switching modes pulsed vertical-field switching (VFS) and pulsed in-plane switching (IPS) are used to enable different texture transitions in the CLC [41]. A perspective view of smart windows and schematic diagram of three stable state switching modes are shown in Figure 12b [103]. Combining the CLC tri-stable state and the dicrotic dye, the smart windows can control the optical performances switching among the transparent (ULH), dark–clear (P, for daylight dimming) and dark scattering (FC, for privacy protection) [35].

Polymer stabilized liquid crystal (PSLC) devices can be used as smart privacy windows that switch between transparent and opaque states. Image of PSLC on PET substrate is off state (Figure 12c) [21]. The image of PSLC on PET substrate is on the state (Figure 12d). The polyimide alignment layer of a PSLC device is usually obtained by the treatment of polyamide acid (PAA) with temperatures over 200 °C. Molecular structure of the chemicals used for the fabrication of polymer-stabilized liquid crystal (PSLC) (Figure 12e) [84].

As a representative polymer dispersed liquid crystal (PDLC) device, little attention has been paid to heat management. Herein, a TiO_2_/Ag (Cu)/TiO_2_ (TCAT) electrode was designed and optimized to achieve the tunability of transmission in the visible range and near-infrared heat shielding simultaneously [105].

The schematic of the fabrication process of the oxide/Ag/oxide electrode-based polymer dispersed liquid crystal (PDLC) device (Figure 12f) [100]. An oxide underlying layer, an oxide upper layer and an ultrathin Ag (Cu) metal intermediate layer were deposited sequentially on PET substrate to form a laminated oxide/Ag/oxide electrode structure. The liquid crystal droplets are then dispersed by ultraviolet curing polymer and sandwiched between the above symmetric oxide/Ag/oxide electrodes to obtain a polymer dispersed liquid crystal (PDLC) device [118]. Two modulation modes in TAT electrode-based PDLC devices are described (Figure 12g). At the OFF state (without applied voltage), the PDLC device strongly scat visible and NIR light due to the random orientation of liquid crystals in the polymer. At the ON state (with applied voltage), owing to the liquid crystal alignment, the visible light is allowed to pass through, while NIR light is partially rejected by TAT electrodes due to its infrared reflection nature [10].

### 4.3. Brief Conclusion

We have introduced the new technology of the multifunctional LCD smart window. Utilizing the different properties of materials, multiple stable and multifunctional liquid crystal smart windows can be obtained, and a new type of low-cost, energy-saving smart windows can be realized.

## 5. Conclusions

In summary, the rapid development of energy-saving smart windows has attracted attention to novel thermochromic materials. This paper systematically summarizes the research progress of smart windows from the perspective of thermochromic with organic materials. For different organic materials, the types, mechanisms and properties of various organic materials are summarized, and the multifunctional smart windows derived from thermochromic technology are discussed. In this section, we will comment on these smart windows and outline challenges and future developments in this field.

Challenges and opportunities remain in the future development of the smart architectural window, with the aim of achieving high energy-saving efficiency. Challenges remain as to how smart windows technology can be fully utilized in buildings in future development. Although some new smart windows have been commercialized, the lack of technology and high costs have prevented their widespread adoption. (1) The cost is too high. Market analysis shows that the additional cost of smart windows comes from installation, maintenance, repair, etc. However, this new organic material can change the way windows are installed and maintained, greatly reducing costs. In addition, to improve the cost performance of the product, the structure and morphology of the material can also be optimized to make its structure more solid and simple and improve the performance of the smart window [97]. (2) Durability. Commercial energy-saving smart windows need high durability to be able to be used for a long time, which has strict requirements for the durability of materials and equipment. At present, the primary task is to improve the structure and optical properties of materials. (3) Maximize energy saving. The key to energy-saving smart windows is to optimize solar modulation. At present, the important parameter to evaluate the control ability of smart windows is sunlight modulation efficiency (∆*T*_sol_) [98,102]. At the same time, to avoid the influence of indoor lighting, the smart window is required to have a higher transmittance in the visible light band (380–780 nm), and the performance is generally evaluated by a visible light transmittance ratio (*T*_lum_) [119].

In future development, more natural organic polymer materials can be used [61]. In traditional synthetic polymers, the cost is too high to degrade easily [101,116,117]. In the face of increasing energy demand, the material selection of smart windows in the future will be more inclined to meet the requirements of the green chemical industry renewable natural organic polymer materials. This will help make better use of organic and inorganic composite materials to effectively combine the advantages and disadvantages between them. Through the synergistic effect, the utilization efficiency of solar energy is further improved, and the structure and morphology of the material are optimized. Thermochromic materials with controllable size and morphology were designed and prepared by optimizing the properties of the materials, and the performance of smart windows was improved by reasonable matching between different materials.

Finally, energy-efficient smart windows hold much promise for contributing to a future, eco-friendly world. However, there is still much work to be done to make energy-efficient smart windows widely available. This summary will attract the interest of more researchers in this field and accelerate the development of energy-saving smart windows through the selection of suitable materials and the integration of technologies.

## Figures and Tables

**Table 1:** Thermochromic properties of 52 μm thick PNIPAm hydrogel films [21].

	20 °C	30 °C	35 °C	40 °C	50 °C	60 °C
*T*_lum_ (%)	87.9	63.3	62.6	59.9	56.5	53.6
*T*_IR_ (%)	80.0	72.6	72.7	70.5	68.4	66.1
*T*_sol_ (%)	83.0	65.5	65.0	62.3	59.8	57.5

**Table 2:** Thermochromic performance of various hydrogel smart windows.

Category	Materials	*T*_lum_ (%)	∆*T*_sol_ (%)	*τ*_c_ (°C)	Characteristic	Ref.
Single function hydrogel	PNIPAm	70.7	25.	32	Conventional thermochromic hydrogel with excellent *T*_lum_, ∆*T*_sol_ and suitable *τ*_c_.	Zhou et al., 2014 [21]
Regular the solar modulation ability	PNIPAm-VO_2_ PNIPAm/VO_2_@ SiO_2_	62.6	34.7	–	By enhancing the IR regulating ability, the ∆*T*_sol_ was increased.	Zhou et al., 2015 [50]
		38.4	62.7	40		Wang et al., 2018 [23]
Photo-thermochromic	ATO/PNIPAm GO/PNIPAm	62.7	35.7	–	High absorbance materials were added to increase the response speed of thermochromic hydrogels.	Lee et al., 2017 [54]
		–	58.2	–		Chou et al., 2017 [57]

“–” means data not available.

**Table 3:** Application of thermal response IL/PILs in smart windows and challenges for future development [99].

Application	Representative Thermally Responsive ILs/PILs	Major Challenges	Refs. [103,104,105,106]
Smart windows **polymeric-IL system** **(Thermochromic)** 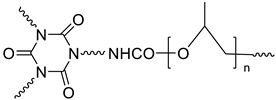 **LCST: 30~35 °C** **Polyurethane network**		Understand and optimize of ILs/PILs-matrix interactions. Improve the sealing methods for IL/PILs.	
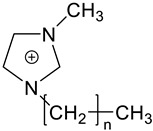 **n = 1, 3, 5** **LCST: 35~40 °C** **[C_n_ mim] cation**		Develop IL/PILs with multi-functionality, wide solar modulation ability durability	
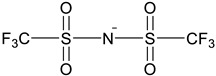 **LCST: 40~41.4 °C** **Thermo- and electro-chromic P(NIPAM–DAV) IL gels**			
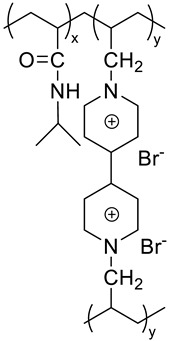 **LCST: 22.8~30.1 °C** **Thermo- and electro-chromic P(NIPAM–BVIm–DAV) gels**			
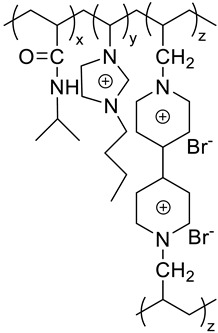 **LCST: 35.2~46.3 °C** **Preparation of Vanadium Oxide Ionogel in scCO_2_/IL biphasic system**			
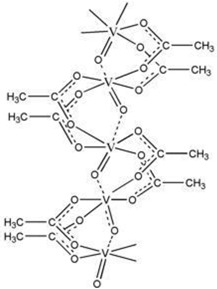 **LCST: 38~40 °C**			

## Data Availability

The study did not report any data.
